# Community-led management maintains higher predator biomass supporting kelp forests persistence in Baja California

**DOI:** 10.1038/s41598-025-86140-6

**Published:** 2025-07-02

**Authors:** Jeremie Bauer, Rodrigo Beas-Luna, Luis Malpica-Cruz, Alicia Abadía-Cardoso, Paulina Filz, Juan Carlos Bonilla, Julio Lorda

**Affiliations:** 1 Departamento de Biotecnología Marina, Centro de Investigación Científica y de Educación Superior de Ensenada, Carretera Tijuana-Ensenada 3918, Fraccionamiento Playitas, 22860 Ensenada, Baja California Mexico; 2 Facultad de Ciencias Marinas, Universidad Autónoma de Baja California, Carretera Tijuana-Ensenada Km 103, Pedregal Playitas, Ensenada, Baja California Mexico; 3 Instituto de Investigaciones Oceanológicas, Universidad Autónoma de Baja California, Carretera Tijuana-Ensenada Km 103, Pedregal Playitas, Ensenada, Baja California Mexico; 4 ECOCIMATI, A.C., Av. Del Puerto 2270 Colonia Hidalgo, 22880 Ensenada, Baja California Mexico; 5 Sociedad Cooperativa de Producción Pesquera La Purísima, Bahía Tortugas, Baja California Sur Mexico; 6 Facultad de Ciencias, Universidad Autónoma de Baja California, Carretera Tijuana-Ensenada Km 103, Pedregal Playitas, Ensenada, Baja California Mexico; 7 Tijuana River National Estuarine Research Reserve, 301 Caspian Way, Imperial Beach, CA USA

**Keywords:** Ecosystem ecology, Climate-change ecology, Macroecology

## Abstract

Community-led management in small-scale fisheries represents an alternative approach to marine ecosystem conservation. This work examines the effectiveness of community-led marine reserves (MRs) by comparing kelp forest canopy coverage and predator populations between two regions with different social-ecological conditions along the Pacific coast of Baja California, Mexico. We analyzed kelp canopy coverage from 2004 to 2023, spanning periods before, during, and after extreme marine heatwaves (2014–2016). Additionally, we compared the density, size, and biomass of three key predator species (spiny lobster, California sheephead, and horn shark) between community-led MRs and federally managed marine protected areas (MPAs). Our analyses revealed significant regional differences in kelp forest coverage recovery following extreme warming events, with the southern region maintaining historical coverage levels while the northern region showed a 95% decline in 2023. Community-led MRs maintained significantly higher densities and biomass of predator species compared to federally managed areas, with spiny lobsters and horn sharks completely absent from northern MPAs and California sheephead showing larger sizes in community-led MRs. These findings demonstrate that community-led MRs maintain predator populations, which may be crucial for future management scenarios given the importance of predator–prey relationships in kelp forest ecosystems. For policymakers, our results suggest that incorporating local governance and community-based approaches into marine management frameworks, particularly in regions with strong fishing cooperatives and traditional management practices, could enhance conservation outcomes. This study provides evidence from the Global South that locally managed, participatory approaches can achieve effective conservation outcomes, offering insights for regions facing similar challenges in balancing resource use with ecosystem protection.

## Introduction

Kelp forests are among the world’s most productive and diverse ecosystems, providing essential habitat, food, and ecosystem services^1,2^. However, kelp forests are increasingly threatened by anthropogenic stressors and climate change impacts^3^. For example, in the past decade, the Northeastern Pacific has experienced multiple concurrent stressors including marine heatwaves, loss of predator species, increased herbivory pressure, and fishing impacts leading to a dramatic decline in kelp forests, with some areas losing more than 90% of their kelp cover and shifting to persistent sea urchin barrens^4,5^.

The recent loss of kelp forests in the Northeastern Pacific is attributed to several key processes. An extreme warming event from 2014 to 2016, one of the most significant and prolonged marine heatwaves (MHWs) ever recorded with severe effects on these ecosystems^6–8^. This warming greatly impacted kelp forests and associated species in Baja California, near the southern distribution limit of *Macrocystis pyrifera*, the primary canopy-forming macroalgae. Species experienced range shifts^9^, kelp forest cover was drastically reduced^4^, and community assemblages shifted towards species with warmer affinities^10^. Additionally, there was a significant mortality event of the sea urchin predator, the sunflower star, *Pycnopodia helianthoides*^11^. Invasive macroalgae began to dominate some reefs^12^, and new predator–prey interactions emerged due to processes such as tropicalization^13,14^. These stressors have caused geographic variability and led to different recovery trajectories in kelp forest communities^15^.

A decade after the first extreme warming event was observed, herbivore grazing, particularly by sea urchins, has been identified as the primary factor preventing the recovery of kelp forests in some regions of the Northeastern Pacific (i.e. central California)^5,16^. Depleting available drifting macroalgae biomass has shifted sea urchins from passive to active feeding behavior^17^. As macroalgae resources diminish, coralline algae cover increases, creating a feedback loop that promotes the establishment and persistence of urchin barrens. This relationship between coralline algae and urchin barrens exacerbates competition with macroalgae for substrate, hindering kelp forest recovery^2^.

In addition to these ecological processes, fishing pressure on macroinvertivores that prey on sea urchins has likely exacerbated the problem^18–21^. For example, in the southern California Bight, Key predators such as the spiny lobster, *Panulirus interruptus*^22^, California sheephead, *Bodianus pulcher*^23^, and the horn shark, *Heterodontus francisci*^24^, play crucial roles in maintaining the balance of kelp forest ecosystems. The decline of these predators due to fishing pressure^21^ and increased temperatures^25^ could reduce the resilience—a system to support key functions after the impact of stressors—of kelp forests, particularly during and after periods of environmental stress^26^.

Kelp forest decline and subsequent community shifts are increasingly documented worldwide, demonstrating similar patterns despite geographic separation. In Tasmania and Aotearoa New Zealand, extreme warming events coupled with range expansion of the sea urchin *Centrostephanus rodgersii* led to the loss of kelp forests, transforming productive ecosystems into persistent urchin barrens, particularly in areas where key predator populations are depleted^27,28^. In central California, the synergistic effects of marine heatwaves and the loss of sea star populations due to wasting disease resulted in widespread kelp forest collapse, particularly affecting bull kelp *Nereocystis luetkeana* populations^5^. These global examples highlight how the interaction between climate stressors and trophic cascade disruptions can lead to rapid ecosystem transformation. Hence, conservation and management strategies (i.e. marine protected areas, territorial user rights for fisheries) will be crucial to enhance future resilience in marine ecosystems.

Establishing Marine Protected Areas (MPAs) has been crucial in conserving and managing marine ecosystems^29^, including kelp forests^30^. Yet, the level of enforcement and surveillance will define the effectiveness of MPAs, particularly related to poaching^31^. Integrating community-based management approaches is a promising solution for more effective conservation strategies. For example, in Aotearoa New Zealand, the establishment of traditional management areas (i.e. Taiāpure reserve) has integrated Indigenous ecological knowledge with modern conservation practices, leading to benefits in fisheries sustainability and ecosystem health^32^. Chilean coastal communities have demonstrated similar success through their Territorial User Rights for Fisheries (TURFs) system, where local fishing organizations manage defined coastal areas, resulting in enhanced resource sustainability and increased biomass of commercially important species^33^. In Hawaii and the Cook Islands, the revival of traditional marine tenure systems has strengthened local governance and improved conservation outcomes^34^. These examples demonstrate how community-led management can effectively balance resource exploitation with ecosystem protection, particularly when supported by strong local governance structures and traditional or local ecological knowledge.

On the Pacific coast of the Baja California peninsula in Mexico, the distribution of resources, users, governance, and private investment varies significantly along the coastline^35^. This variability provides a unique opportunity to study a latitudinal gradient (~1000 km) for ecological patterns in response to environmental and socioeconomic factors. Moreover, in Mexico and the Baja California peninsula, federally managed MPAs, fishing refuges, and state reserves exist, with different goals and varying levels of protection and enforcement^36^. Specifically, while including marine areas within their decree polygons, the Vizcaíno Biosphere Reserve (in the southern region of our study) and the Baja California Pacific Island Biosphere Reserve (in the northern region of our study) are not considered fully protected no-take zones. Their mandates and management plans are defined as buffers, or core zones, where certain low-impact activities are allowed. While extensive mining and industrial fishery activities are prohibited, artisanal fisheries have concession zones and permits to harvest different benthic invertebrates, algae, and fish species.

Noteworthy are community-led no-take marine reserves (MRs) that have been designed, implemented, and promoted for over 80 years in Baja California’s temperate reefs^37,38^. These MRs have demonstrated the potential for adequate protection and recovery of economically important key species such as abalone^39–42^. These MRs, where communities allocate a portion of their fishing grounds for protection and actively engage in surveillance, have succeeded in areas with TURFs, referred to in Mexico as concession zones, and where the fishing cooperatives are actively involved in the management and governance of their fisheries^37,43,44^.

Our study examines the temporal patterns of *M. pyrifera* canopy coverage along the Pacific Coast of Baja California spanning two decades (2004–2023), including an extreme warming event (2014–2016). We compared two regions with different management approaches—community-led management in the south and federally managed partially protected MPAs in the north with multiple stakeholders fishing for resources- focusing on three key ecological and commercially important predator species: spiny lobster (*P. interruptus*), California sheephead (*B. pulcher*), and horn shark (*H. francisci*). Specifically, we (1) analyzed changes in kelp canopy coverage before, during, and after the extreme warming event in both regions; (2) compared the abundance, size, and biomass of these three predator species between protected and fished areas; and (3) examined potential relationships between management approach and these ecological indicators. While our study focuses on a subset of predator species rather than the entire kelp forest community, our findings provide insights into how different management strategies may influence key predator populations following environmental disturbance. This research contributes to our understanding of marine management approaches in the Global South, particularly in regions where community-led and traditional management practices coexist with federal management schemes.

## Materials and methods

### Study area

**Fig. 1:**
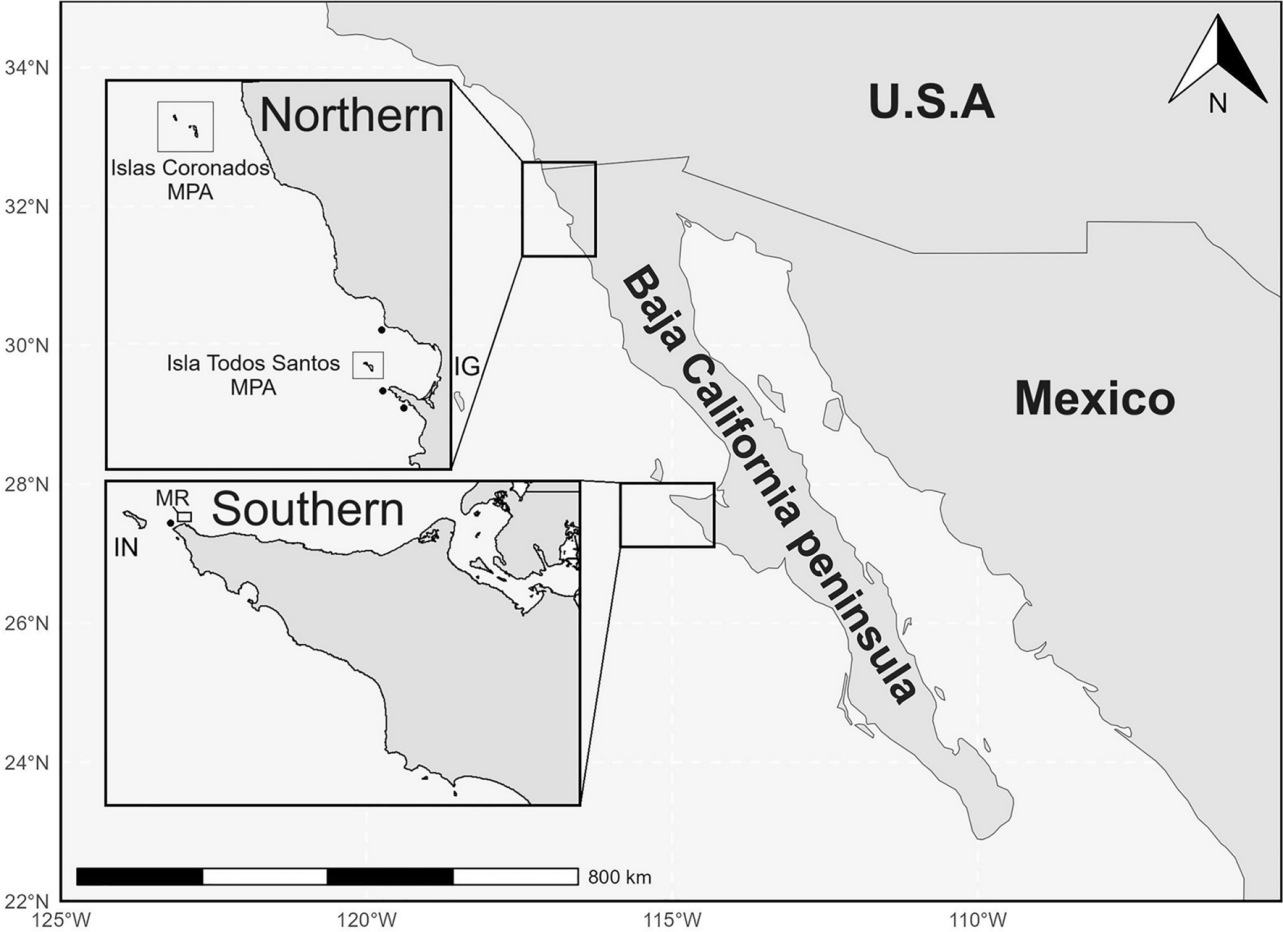
Study regions along the Pacific coast of Baja California, Mexico. The northern region includes two federally managed Marine Protected Areas (MPAs): Islas Coronados and Isla Todos Santos. The community-led marine reserve (black box, MR) is in the southern region. Black circles indicate monitored fished sites in both regions. IN = Isla Natividad; IG = Isla Guadalupe. The map was generated using R version 4.3.1 (R Core Team, 2024) with the maps package version 3.4.4 and ggplot2 version 3.4.4.

Our study encompassed two regions along the Pacific coast of Baja California, Mexico (Fig. 1). In the northern region, we monitored sites within two federally managed partially protected MPAs: Islas Coronados and Isla Todos Santos. In the southern region, we monitored a community-led MR called Piedra Blanca. Our sampling design included fished sites (black circles in Fig. 1) in both regions, allowing comparison between protected and unprotected areas. While not directly monitored in this study, Isla Guadalupe and Isla Natividad (Fig. 1) provide important context discussed later.

The southern region is the distribution limit of *M. pyrifera* in the northeastern Pacific^45^. The interplay between the California Current and the Davidson Current^46^ influences the oceanographic dynamics of this region. The California Current flows southward along the coast, bringing cold, nutrient-rich water from the North Pacific^47^, which supports high primary productivity and the growth of kelp forests. Conversely, the Davidson Current flows northward during the fall and winter, introducing warmer, less nutrient-rich water from the south^46^. The convergence of these two currents creates a transition zone that contributes to the southern region’s unique biodiversity and ecological processes^48^.

Throughout the study area, complex social-ecological dynamics are affecting the sustainability of fishery systems. In particular, the northern region faces fishery management challenges^35^ and illegal fishing activities that significantly threaten the sustainable use of several marine species^49,50^. On the other hand, the southern region has demonstrated successful fishery management practices, with well-organized fishing cooperatives and community-based conservation initiatives^35,37,51^. For example, community-led and community-enforced no-take MRs are a consistent strategy used by the fishing cooperatives in the area^37,41,42^.

### *Macrocystis pyrifera* canopy coverage and sea surface temperature assessment

We assessed kelp canopy coverage and sea surface temperature (SST) using satellite imagery data^52^. We then processed Landsat satellite images to estimate the extent of kelp canopy (m^2^) and SST (ºC) in the study regions. The images were acquired quarterly, providing four annual measurements to capture the seasonal variability in kelp coverage. The quarterly data were averaged to obtain yearly mean kelp canopy coverage values for each region (northern and southern) from 2004 to 2023. Finally, we assessed monthly means for the SST.

We performed a relative change analysis to assess kelp canopy coverage area changes relative to each region and year’s historical mean (2004–2013). Then, for each region and year from 2004 to 2023, we calculated the percentage change by subtracting the historical mean from the observed kelp canopy coverage area, dividing the result by the historical mean, and multiplying by 100. This analysis provides insights into the magnitude and direction of kelp canopy coverage area changes relative to the historical baseline. Positive values indicate an increase in kelp canopy area compared to the historical mean, while negative values indicate a decrease.

### Macroinvertivores monitoring and reserve protection

We conducted underwater surveys through SCUBA diving. At each monitoring site, 30 m transects were deployed parallel to the coastline in depths ranging from 5–18 m. Divers surveyed a 2 m wide belt along each side of the transect line, resulting in a total monitored area of 60 m^2^ per transect. From 2016 to 2022, we deployed 175 transects in the northern region, with 89 transects inside partially protected MPAs (Islas Coronados and Isla Todos Santos) and 86 transects in three fishing sites outside MPAs (Campo Kennedy, Punta Banda, and San Miguel). In the southern region, we deployed 152 transects, 75 transects inside a community-led MR (Piedra Blanca) and 77 transects outside the MR in a fishing ground (Gavilanes). Along each transect, we recorded the abundance of three ecological and economically important macroinvertivores species shared between the two regions: the spiny lobster (*P. interruptus*), the California sheephead (*B. pulcher*), and the horn shark (*H. francisci*).

We used the density (individuals m^-2^) of the three macroinvertivore species for the analyses. To further investigate biomass differences between regions, we used the length–weight relationship for California sheephead^19^: W = 0.0144 × TL^3.04^, and horn shark^53^: W = 0.006 × TL^3.06^. Where W is the weight in grams, and TL is the total length in centimeters estimated during monitoring. We first calculated each region’s mean size and SE using size and frequency data. The data consisted of unique size values in centimeters, while the frequency data indicated the number of individuals observed at each size. To account for the frequency of each size class, we created a list of weighted sizes by repeating each value based on its corresponding frequency. For lobsters, we estimated the biomass based on Supplementary Table 1.

### Statistical analyses

We performed statistical analyses to assess the differences in kelp canopy coverage between the northern and southern regions and to examine the impact of the extreme warming event on kelp coverage. We used a Mann–Whitney test to compare kelp coverage between the two regions with data from 2004–2023. Then, we conducted Kruskal–Wallis tests for each period (before = 2004–2013, during = 2014–2016, and after = 2017–2023 extreme warming events) to determine if there were significant differences in kelp coverage between regions within each period. We performed Wilcoxon rank-sum tests to investigate the pairwise differences between regions for each period. We adjusted the p-values from these tests using the Benjamini–Hochberg method to control for multiple comparisons.

For each period, we calculated the mean monthly SST (± SE) to assess regional differences in temperature regimes and the magnitude of warming during the warming events. Due to the non-normal distribution of the temperature data, we used non-parametric tests to examine differences between regions and periods. Kruskal–Wallis tests were used to examine differences in SST across the three time periods within each region. Mann–Whitney U tests were used to compare temperatures between regions within each period.

To analyze the effects of region (northern vs. southern) and reserve status (inside vs. outside) on the density and biomass of *P. interruptus*, and the density, size, and biomass of *B. pulcher* and *H. francisci*, we employed different statistical approaches based on the nature of the data. For *P. interruptus*, we used a zero-inflated negative binomial model due to the high occurrence of zero counts, particularly in the northern region. This model accounts for the excess zeros and the overdispersion in the count data. We interpreted the coefficients and their significance to assess the effects of region and reserve status on lobster abundance. For *H. francisci* and *B. pulcher*, we employed the Scheirer-Ray-Hare test, a non-parametric alternative to the two-way ANOVA, because the data did not meet the assumptions of normality and homogeneity of variances. We conducted separate tests for presence/absence data, size data (where present), and biomass data. We included all transects for presence/absence analysis, treating zero observations as meaningful ecological data. We only considered transects where the species was present for size analysis, as size cannot be measured for absent individuals. We included all transects for biomass analysis, treating zero biomass as ecologically significant information. We included the interaction term between region and reserve status in all models to assess whether the effect of reserve status differed between regions. Significance was determined at α = 0.05. Where significant effects were found, we conducted post-hoc pairwise comparisons using Dunn’s test with Bonferroni correction for multiple comparisons. All statistical analyses were performed using R (version 4.3.1; R Core Team, 2024).

## Results

All sampling sites were located within two distinct regions separated by approximately 500 km along the Baja California peninsula (Fig. 1). This geographic separation, combined with different management approaches—federal partially protected MPAs in the north with multiple stakeholders versus community-led management with a MR in the south—provided a natural experiment to examine the interaction between environmental impacts and protection status.

### Changes to canopy coverage of giant kelp, *M. pyrifera*

**Fig. 2:**
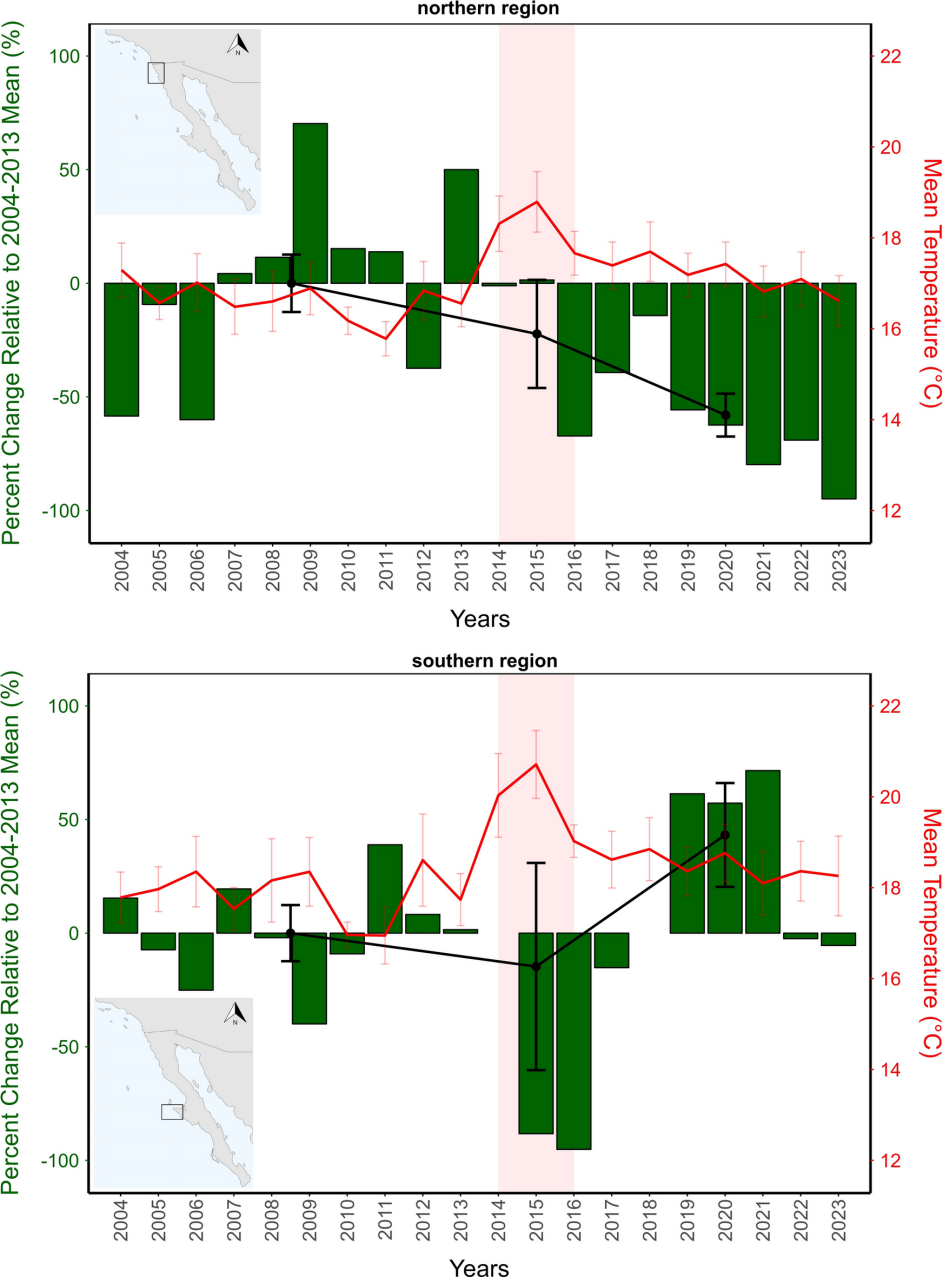
Giant kelp, *Macrocystis pyrifera*, percent change relative to the 2004–2013 historical mean (%) before the extreme warming event (highlighted in red) for the northern and southern regions in the left y-axis. The black line indicates the average ± SE of the historical mean (2004–2013), the extreme warming event (2014–2016), and after the warming event (2017–2023). The red line indicates the average ± SE of the sea surface temperature for both regions in the right y-axis.

Temporal and spatial patterns of *M. pyrifera* canopy coverage area differed significantly between the northern and southern regions of the study area (Fig. 2). The Mann–Whitney test revealed a significant difference in kelp coverage between the two regions (W = 2445, p = 0.01891). In the northern region, kelp coverage decreased from 2004 to 2023. The mean average (± SE) canopy of 2023 (124,262 ± 101,124 m^2^) shows a 94.9% decrease compared with the 10-year historical mean 2004–2013 (2,427,154 ± 306,768 m^2^) before the extreme warming events. In contrast, the southern region showed a relatively stable trend in kelp coverage throughout the study period, with notable declines during the extreme warming events of 2014–2016, followed by an increase after the warming events (Fig. 2). In the southern region, the mean average canopy of 2023 (2,579,857 ± 1,015,915 m^2^) is only 5.41% lower than historical mean 2004–2013 (2,727,451 ± 336,915 m^2^) before the warming events.

The Kruskal–Wallis tests conducted for each period (before, during, and after the warming events) indicated significant differences in kelp coverage between the northern and southern regions during (p = 0.0117) and after the warming events (p < 0.001). Still, we found no differences before the warming events (p = 0.413). Pairwise Wilcoxon rank-sum tests further confirmed these findings, revealing significant differences between the regions during (p = 0.0104) and after (p < 0.001) warming periods. Again, no significant difference was found before (p = 0.418) the warming events period.

Sea surface temperatures differed significantly between regions and across periods. The southern region consistently maintained warmer temperatures than the northern region across all periods (Mann–Whitney tests, p < 0.001 for all periods). Prior to the marine heatwave (2004–2013), mean SST was 16.97 ± 0.12 °C in the northern and 18.21 ± 0.14 °C in the southern region. During the marine heatwave (2014–2016), both regions experienced significant warming (Kruskal–Wallis tests, northern: H = 42.89, p < 0.001; southern: H = 38.76, p < 0.001), with mean temperatures increasing to 18.54 ± 0.21 °C in the northern and 20.28 ± 0.24 °C in the southern region. Following the heatwave (2017–2023), temperatures moderated but remained elevated compared to pre-warming conditions, with means of 17.45 ± 0.13 °C and 19.12 ± 0.15 °C in the northern and southern regions, respectively.

### Macroinvertivores densities, size, and biomass

**Fig. 3:**
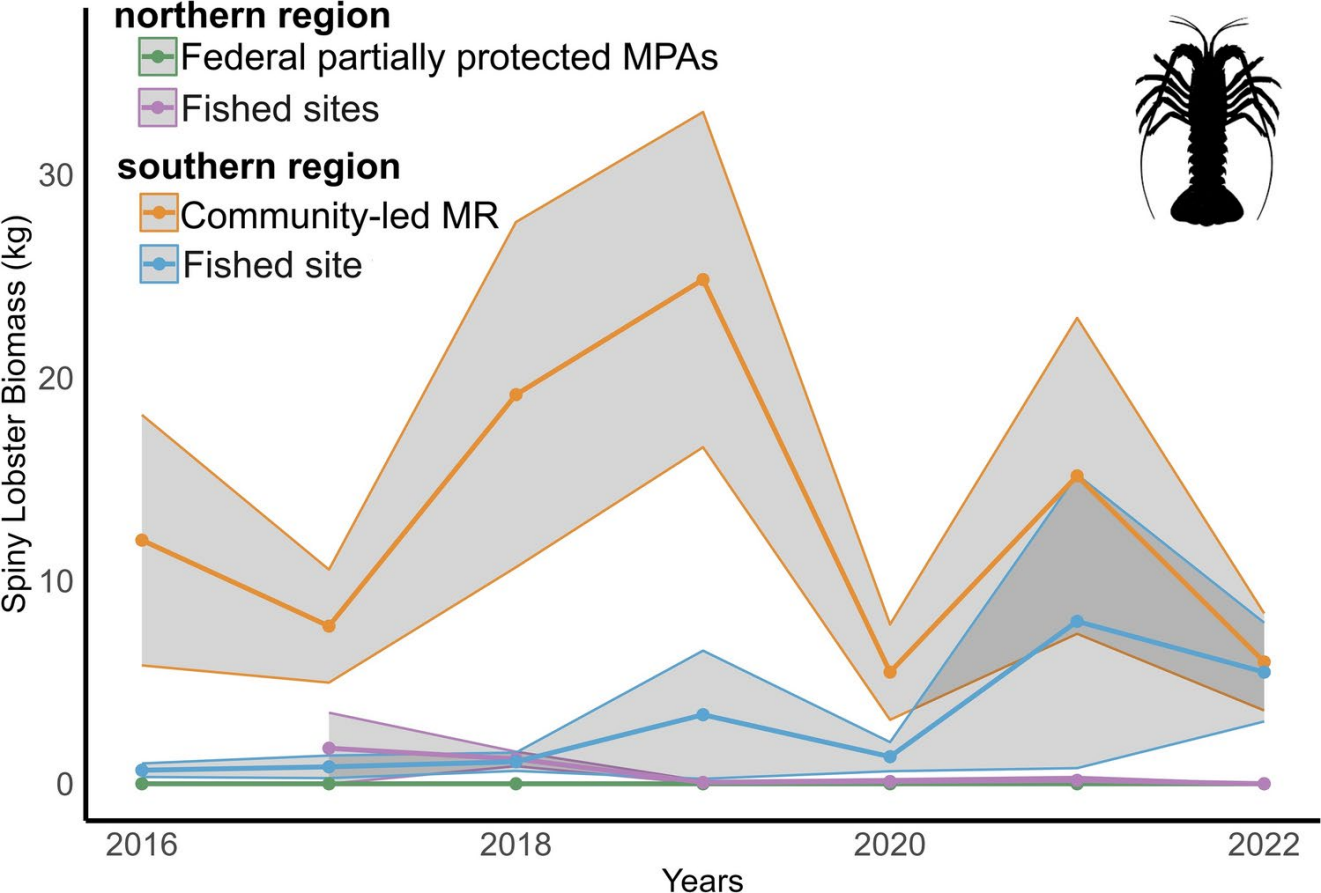
Annual biomass (kg) of spiny lobsters, *Panulirus interruptus*, from 2016 to 2022. Lines represent the mean biomass, and shaded areas represent the SE for each region and reserve status. The green line represents the northern region partially protected MPAs (n = 89) and the purple line fishing grounds (n = 86). The southern region orange line represents the community-led MR (n = 75) and the blue line fishing grounds (n = 77). MPA = marine protected area; MR = no-take marine reserve.

The mean ± SE densities per transect of *P. interruptus* across years in the northern region was 0.006 ± 0.01 ind m^-2^ (0 inside partially protected MPAs, 0.01 ± 0.02 in fished sites). In the southern region, the mean density was 0.12 ± 0.02 ind m^-2^ (0.20 ± 0.02 inside the MR, 0.04 ± 0.02 in the fished site). This resulted in a mean ± SE biomass per transect in the northern region of 0.26 ± 0.16 kg (0 inside partially protected MPAs and 0.55 ± 0.30 in fishing sites). In the southern region, the mean biomass was 7.95 ± 1.98 kg (12.90 ± 2.74 inside the MR and 2.97 ± 1.07 in the fishing site) (Fig. 3).

The zero-inflated negative binomial model revealed significant effects for regions (β =  − 0.7621, p < 0.001), reserve status (β =  − 0.3506, p < 0.001), and their interaction (β = 0.6242, p < 0.001). In Table 1, the intercept represents the baseline condition (southern region, fished sites). The region term compares northern to southern regions, the protection status term compares reserves to fished areas, and their interaction tests whether the effect of protection differs between regions. Negative estimates indicate lower density relative to the baseline, while positive estimates indicate higher density. Post-hoc comparisons showed significant differences between the northern region inside MPAs and all other categories (p < 0.001 inside and outside the MR in the southern region, p = 0.0004 for northern fishing grounds), as well as between inside the southern MR compared to northern fishing grounds (p = 0.0019) (Supplementary Table 2).

**Table 1:** Zero-inflated negative binomial model results testing the effects of region and protection status on lobster (*P. interruptus*) density.

Factor	Estimate	Std. Error	z value	Pr( >|z|)
Intercept (southern region, fished sites)	1.02508	0.05473	18.731	2e^−16^
Region (northern vs southern)	− 0.76209	0.10821	− 7.043	1.88e^−12^
Reserve (reserve vs fished)	− 0.35063	0.0895	− 3.918	8.94e^−05^
Region × Protection interaction	0.62417	0.14825	4.21	2.55e^−05^

**Fig. 4:**
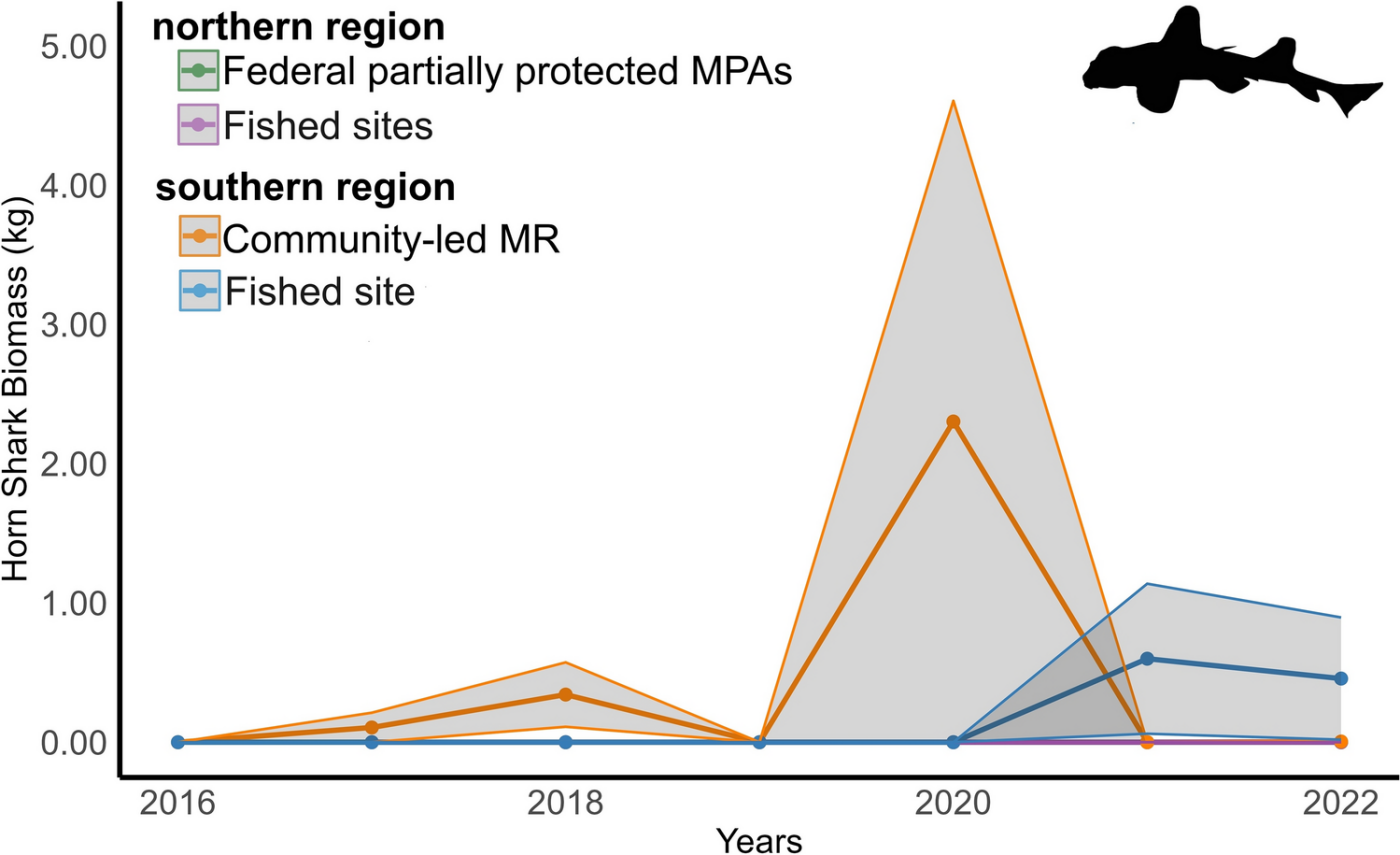
Annual mean biomass (kg) of the horn shark, *Heterodontus francisci*, from 2016 to 2022. Lines represent the mean biomass, and shaded areas represent SE for each region and reserve status. The green line represents the northern region partially protected MPAs (n = 89) and the purple line fishing grounds (n = 86). The southern region orange line represents the community-led MR (n = 75) and the blue line fishing grounds (n = 77). MPA = marine protected area; MR = no-take marine reserve.

For *H. francisci,* the mean ± SE densities per transect in the northern region was consistently 0 across all monitoring years inside or outside the partially protected MPAs. In the southern region, the density was 0.001 ± 0.0003 ind m^-2^ (0.001 ± 0.0004 in the MR and 0.001 ± 0.0004 in the fished site). The mean ± SE size in the southern region was 61.3 ± 9.43 cm (61.3 ± 12.8 inside the MR and 61.2 ± 16.0 in the fished site). This resulted in a mean ± SE biomass per transect of 0 in the northern region and 0.216 ± 0.114 kg in the southern region (0.248 ± 0.181 inside the MR and 0.179 ± 0.129 in the fished site) (Fig. 4). The Scheirer-Ray-Hare test showed a significant effect of region on *H. francisci* density (H = 10.5246, p = 0.00118), Size (H = 11.4916, p = 0.0007), and biomass (H = 10.5272, p = 0.0011) but no significant effect of reserve status nor interaction between region and reserve status (Table 2). Post hoc significant comparisons are available in Appendix Table 2.

**Table 2:** Scheirer–Ray–Hare test results for *B. pulcher and H. francisci*, density, size, and biomass.

Species	Measure	Factor	Df	Sum Sq	H	p-value
*B. pulcher*	Density	Region	1	0.1917	29.086	< 0.001
		Reserve	1	0.0167	2.539	0.112
		Region: Reserve	1	0.1838	27.889	< 0.001
	Size	Region	1	1,480,767	45.746	< 0.0001
		Reserve	1	13,829	0.427	0.5134
		Region: Reserve	1	2199	0.068	0.7944
	Biomass	Region	1	471,943	12.4243	0.0004
		Reserve	1	34,387	0.9053	0.3414
		Region: Reserve	1	105,204	2.7696	0.0961
*H. francisci*	Density	Region	1	7151	10.5246	0.0012
		Reserve	1	6	0.0087	0.9256
		Region: Reserve	1	7	0.0101	0.9201
	Size	Region	1	8765	11.4916	0.0007
		Reserve	1	95	0.1264	0.7222
		Region: Reserve	1	109	0.1448	0.7536
	Biomass	Region	1	7155	10.5272	0.0012
		Reserve	1	5	0.0072	0.9325
		Region: Reserve	1	6	0.0083	0.9278

**Fig. 5:**
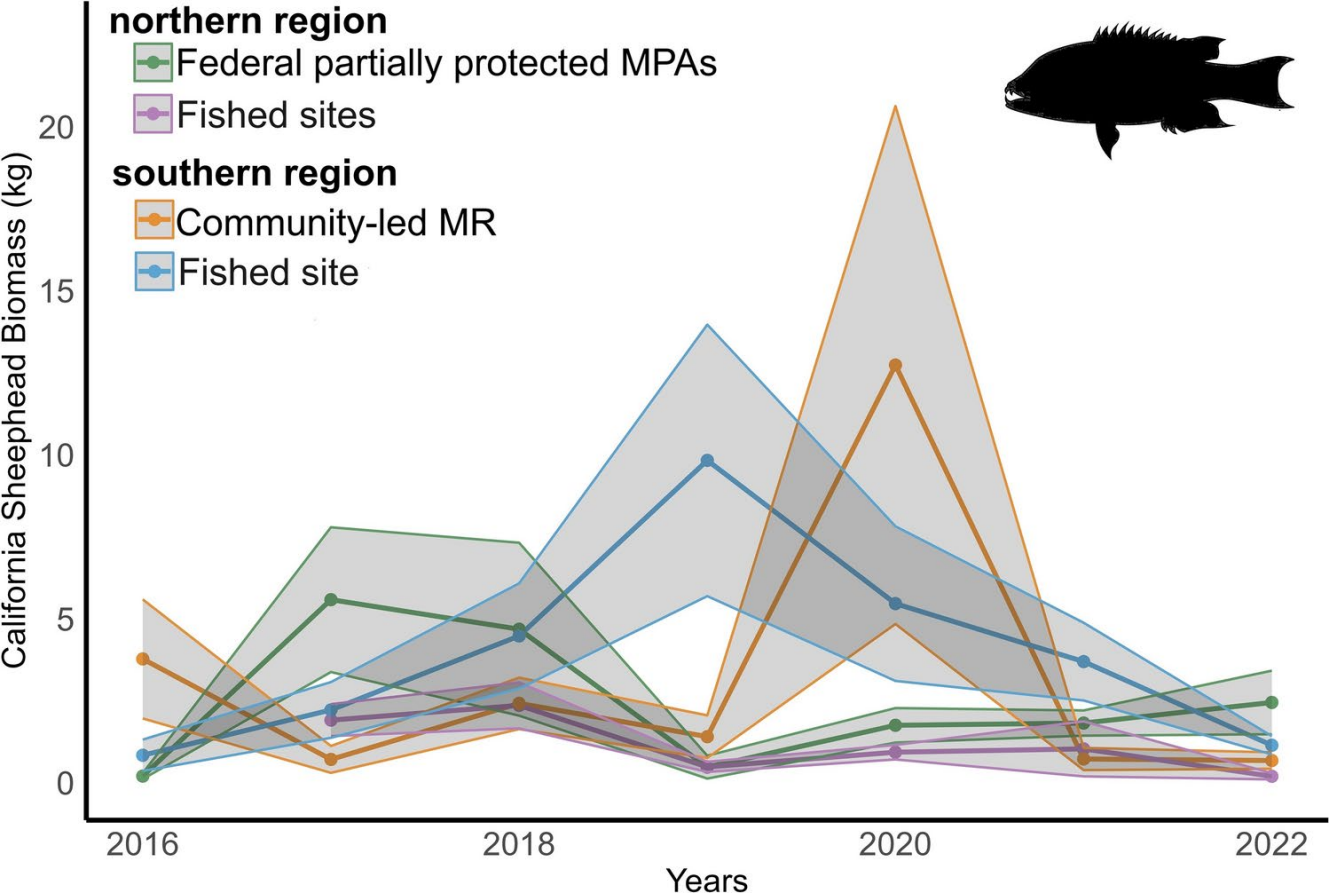
Annual mean biomass (kg) of the California sheephead, *Bodianus pulcher*, from 2016 to 2022. Lines represent the mean biomass, and shaded areas represent the SE for each region and reserve status. The green line represents the northern region partially protected MPAs (n = 89) and the purple line fishing grounds (n = 86). The southern region orange line represents the community-led MR (n = 75) and the blue line fishing grounds (n = 77). MPA = marine protected area; MR = no-take marine reserve.

For *B. pulcher*, the mean ± SE density in the northern region was 0.107 ± 0.007 ind m^-2^ (0.135 ± 0.010 at the partially protected MPAs and 0.077 ± 0.009 at the fished sites). In the southern region, the mean density was 0.057 ± 0.008 ind m^-2^ (0.040 ± 0.012 at the MR and 0.073 ± 0.011 at the fished site). However, in the southern region, the mean total length was 30.10 ± 0.89 cm (30.28 ± 1.28 at the MR and 29.95 ± 1.26 at the fished site), while in the northern region, 20.86 ± 0.88 cm (20.44 ± 1.21 inside MPAs and 21.27 ± 1.20 at fished sites). This resulted in a mean biomass per transect in the southern region of 2.90 ± 0.41 kg (2.45 ± 0.50 inside the MR and 3.34 ± 0.59 at the fished site), and 1.80 ± 0.39 kg in the northern region (2.28 ± 0.54 inside MPAs and 1.26 ± 0.59 kg at fished sites) (Fig. 5).

The Scheirer-Ray-Hare test revealed significant effects of the regions on *B. pulcher* density (H = 29.086, p < 0.001), size (H = 52.72, p < 0.001), and biomass (H = 12.4243, p = 0.0004). A significant interaction between region and reserve status was observed for density (H = 27.889, p < 0.001) but not for size or biomass. Reserve status alone did not show significant effects on any measure. Post hoc Dunn’s tests showed a significant difference between northern fishing grounds and southern MR (p = 0.0494) for density. Size comparisons revealed significant differences between all northern-southern pairs (p < 0.001), with larger sizes in the southern region (Supplementary Table 2).

## Discussion

We suggest that community-led MRs positively affect the small-scale fisheries of key macroinvertivores associated with kelp forests that are impacted by extreme warming events. Our results highlight how poor fishing management could lead to devastating effects on the biomass of targeted organisms, as observed in our study area’s northern federal partially protected MPAs and fishing grounds. In addition, we show how the different levels of protection, enforcement, and local governance within MPAs and MRs could lead to more comprehensive solutions for the resilience of coastal ecosystems worldwide.

Our results revealed striking differences in the canopy coverage recovery of *M. pyrifera* at the regional scale, seven years after extreme warming events on the Pacific coast of the Baja California peninsula, Mexico. While the southern region has shown remarkable resilience following the 2014–2016 warming events, with *M. pyrifera* recovering to historical levels, the northern region has experienced a staggering 94.9% decrease in kelp coverage in 2023 compared to the historical mean (2004–2013) before the warming events. This disparity suggests that factors beyond direct or associated temperature impacts—e.g., nutrient uptake, [O2], pH, etc.- such as herbivory pressure^17^, lack of predators^21^, or competition^12^ contribute to the continued decline of kelp forests in the northern region of Baja California.

Our findings emphasize the importance of considering spatial heterogeneity when assessing the impacts of climate change on kelp forest ecosystems. The significant differences between regions during and after the warming events highlight the potential influence of regional factors, such as oceanographic conditions, ecological interactions, and socioeconomic challenges, on kelp forest dynamics in response to extreme climatic events. The contrasting recovery patterns of kelp coverage in the northern and southern regions suggest the need for region-specific management strategies and conservation efforts to mitigate the effects of warming events on these vital marine habitats.

Sea urchin predators, particularly *P. interruptus* and *B. pulcher*, play a vital role in mediating the resilience of kelp forests to extreme warming events in the southern California region^19,21,54^. In locations where fisheries target these predators, evidence of trophic cascade effects where sea urchins are released from predation with indirect impacts on macroalgae density has been documented^55,56^. Alternatively, within fully protected MPAs that harbor higher abundances of lobsters and sheepheads, stronger top-down control of sea urchin populations has been reported^21^. Evidence indicates that harvesting sea urchin predators in Baja California before the 2014–2016 warming events reduced the population density across the region^25^. Consequently, kelp forests within fully protected areas could exhibit greater resistance and recovery from extreme warming than those in partially protected or unprotected areas. This finding illustrates the consequences of fishing pressure on these key predators, compromising the resilience and persistence of kelp forests to climate-driven disturbances.

In our study, the vast regional differences in densities of *P. interruptus* suggest contrasting fishery management effectiveness between the southern community-led MR and the northern partially protected MPAs. Lobster densities in the southern MR were significantly higher (0.20 ± 0.02 ind m^-2^) than in the northern partially protected MPAs (0 ind m^-2^), where no lobsters were detected across seven years of monitoring. These higher densities in the MR suggest that localized, community-driven management practices may offer superior protection and sustainability for marine species compared to the more complex and less cohesive strategies employed in the northern MPAs. Indeed, MRs in Baja California have been shown to boost biomass productivity of different taxa of economic value^43^.

We observed the same tendency with the horn shark *H. francisci*, a ubiquitously distributed macroinvertivore of kelp forest communities of the Baja California peninsula^57^. While horn sharks are present at low densities in the southern MR (0.001 ± 0.0003 ind m^-2^), their presence is consistent across years. Again, no horn sharks were observed in the northern partially protected MPAs over seven years of monitoring. This is possibly related to fishing pressure, as horn sharks are one of the most caught elasmobranchs by gillnets in small-scale fisheries of northwestern Mexico^58^. This is particularly problematic because of their potential role as overgrazing control^59^ and the possible effect on trophic cascades promoting kelp forest resilience and persistence.

Although sheephead, *B. pulcher*, appear more abundant in the northern sites, these individuals are generally smaller, possibly related to fishery targeting large adults. In contrast, the southern MR hosts bigger sheepheads (30.28 ± 1.28 cm) than the MPAs of the north (20.44 ± 1.21 cm). This difference in size is crucial, as larger sheepheads can consume bigger sea urchins^19^, exerting stronger top-down control on urchin populations. A study by Hamilton & Caselle^19^ demonstrated that the proportion of sea urchins in *B. pulcher* gut contents increased significantly with fish size, from an average of 2.8% in fish less than 200 mm to 38.4% in fish greater than 450 mm. This size-based predation effect is particularly important in areas where lobster populations are depleted, as recently documented in the southern California Current, where sheephead may serve as the primary predator controlling urchin populations in the absence of spiny lobsters^54^. Therefore, the recovery of size structure and biomass of sea urchin predators within well-managed MRs can enhance the resilience of kelp forests by controlling herbivore populations and preventing overgrazing, ultimately supporting the recovery of kelp forests following climate-driven disturbances^4,15,60,61^.

Community MRs or core no-take areas within MPAs could facilitate kelp forest recovery by restoring trophic interactions, such as recovering sea urchin predators. However, the natural recovery process from a sea urchin barren state to kelp forests—even within fully protected MPAs—can take years, even decades^62–64^. Effective management and enforcement are essential to realize the potential benefits of MRs and MPAs. The Baja California Pacific Islands Biosphere Reserve, established in 2016, offers a valuable opportunity to design and implement comprehensive management strategies addressing climate change impacts and fishery management. Yet, this large spanning Biosphere Reserve must include no-take core areas or act synergistically with fishing communities to establish additional MRs within their boundaries to enhance the recovery of meso and top predators^43^. This natural control strategy should increase predation on grazing herbivores, particularly sea urchin populations, which could affect the stability and recruitment of kelps, given the proven devastating effects on kelps at high densities^5,21^. Moreover, the Baja California Pacific Islands Biosphere Reserve lacks marine core no-take zones within the current kelp distribution region from Punta Eugenia to Islas Coronado, Baja California.

One potential option is a multi-faceted approach to safeguard Baja California’s kelp forests and associated fisheries, extending beyond the mere establishment of MPAs. While MPAs are essential conservation tools, their effectiveness is related to various factors, including surveillance and enforcement^65,66^. The success of community-led no-take MRs, such as in Isla Natividad^39,42^ and Isla Guadalupe^40^, demonstrates the potential of participatory conservation approaches. By engaging local fishing communities in protecting and managing their resources, these reserves have achieved effective conservation outcomes and enhanced the resilience of key species like abalone to fishery pressure and extreme environmental events.

Nonetheless, it is crucial to recognize that the success of MRs in Isla Natividad and Isla Guadalupe may be partly attributed to the low number of inhabitants and the isolation of these islands from major urban centers. These factors can facilitate the effective implementation and enforcement of protection and conservation measures in local fishing communities. However, when considering the use of MRs in Isla Todos Santos and Islas Coronados, it is crucial to account for the potential challenges posed by their proximity to large cities (Tijuana and Ensenada) and multiple stakeholders with diverse interests^35^. Nonetheless, the lessons learned from Isla Natividad and Isla Guadalupe can inform the development of adaptive, context-specific approaches that engage local communities and stakeholders in the protection and sustainable use of marine resources in developing countries such as Mexico.

Given the dire condition of kelp forest ecosystems in Baja California, action is warranted. As climate change continues to pressure marine ecosystems worldwide^67^, we must prioritize preserving and restoring key habitats like kelp forests. The Baja California peninsula, with its unique oceanographic setting and diverse stakeholder groups, has the potential to serve as a model for effective, collaborative approaches to marine ecosystem management. The region can build resilience in unprecedented environmental challenges by embracing adaptive nature-based solutions and fostering cross-sectoral partnerships.

While our study demonstrates regional differences in kelp canopy coverage and predator populations, the mechanisms driving these patterns likely involve complex ecological interactions that require further investigation. The higher abundance and larger size of predators in community-led MRs suggest their potential importance for future management scenarios, particularly if herbivory pressure is a key factor preventing kelp recovery. However, understanding the complete picture requires a deeper examination of the entire kelp forest community structure and how it has shifted following extreme warming events. Future research should focus on quantifying regional differences in herbivore populations, particularly purple sea urchins, and their interactions with predator populations. Such comprehensive community-level analyses will be crucial for understanding regional recovery trajectory mechanisms and developing management strategies. This work is a step in understanding how different management approaches affect key species while highlighting the need for broader community-level investigations to fully inform kelp forest conservation efforts.

In conclusion, the health and stability of kelp forests are deeply influenced by climate change and human activities. The stark contrast in management outcomes between Baja California’s northern and southern regions is a reminder of the importance of implementing sound, community-based, effective fishery management, and the need to foster comprehensive and adaptive nature-based solutions. The observed differences between the community-led MR and federal partially protected MPAs likely result from a combination of social-ecological factors, including management approach, geographic isolation, and enforcement capability. Hence, community-led management represents one component of successful conservation, particularly when combined with favorable social-ecological conditions. As we address the ongoing decline of kelp forests in the region and beyond, we must be guided by collaboration, innovation, and a shared commitment to the long-term sustainability of our marine resources and their ecosystem provisioning.

## Supplementary Information

Supplementary Information 1.

Supplementary Information 2.

## Data Availability

The data supporting this study’s findings are available at this online repository (https://github.com/rbeas/MexCal). As the data comes from a monitoring program with constant updates and particular restrictions, access to the repository is available upon request. Contact the corresponding authors at rbeas@uabc.edu.mx or jlorda@uabc.edu.mx to request access to the data repository.
