# Supplementary material for: Community-led management maintains higher predator biomass supporting kelp forests persistence in Baja California
Source: Sci Rep. 2025 Jul 2;15:23253. doi: 10.1038/s41598-025-86140-6 (PMC12222674; doi:10.1038/s41598-025-86140-6)
Supplement: Supplementary file 1 — Supplementary Information 1. [file 41598_2025_86140_MOESM1_ESM.docx]

**Supplementary information**

Supplementary table 1*.* Mass of Fishes and Invertebrates used for biomass estimation in Woodson, C. B. et al. Harnessing marine microclimates for climate change adaptation and marine conservation. *Conserv. Lett.* 12, e12609. <https://doi.org/10.1111/conl.12609> (2019).

| **Fish Species** | **Avg Mass (kg)** | **Invertebrate Species** | **Mass (kg)** |
| --- | --- | --- | --- |
| *Anisotremus davidsoni* | 0.50 | Anemone *spp.* | 0.25 |
| *Caulolatilus princeps* | 3.0 | *Cancer spp.* | 0.50 |
| *Chromis punctipinnis* | 0.10 | *Centrostephanus coronatus* | 0.40 |
| *Embiotica jacksoni* | 0.20 | *Crassedoma giganteum* | 0.25 |
| *Girella nigricans* | 1.50 | *Cypraea spp.* | 0.25 |
| *Halichoeres semicinctus* | 0.10 | *Haliotis spp.* | 0.25 |
| *Heterodontus francisci* | 4.00 | *Kelletia kelletii* | 0.25 |
| *Hypsypops rubicundus* | 0.30 | *Leptogorgia chilensis* | 0.25 |
| *Mycteroperca spp.* | 3.00 | *Loxorhyncus grandis* | 1.00 |
| *Ophiodon elongatus* | 3.50 | *Megastraea turbanica* | 0.50 |
| *Oxyjulis californica* | 0.05 | *Megastraea undosum* | 0.30 |
| *Paralabrax spp.* | 0.80 | *Megathura crenulata* | 0.30 |
| *Rhacochilus vacca* | 0.10 | *Mesocentrotus franciscanus* | 0.25 |
| *Rhinobatos productus* | 8.00 | *Muricea spp.* | 0.25 |
| *Scorpaenichthys marmoratus* | 3.50 | *Neobernaya spadicea* | 0.10 |
| *Sebastes spp.* | 1.00 | *Octopus spp.* | 1.00 |
| *Semicossyphus pulcher* | 3.50 | *Panulirus interruptus* | 1.00 |
| *Squatina californica* | 5.00 | *Patiria miniata* | 0.40 |
| *Stereolepis gigas* | 8.00 | *Pisaster giganteus* | 1.00 |
|  |  | *Pycnopodia helianthoides* | 1.00 |
|  |  | *Strongylocentrotus purpuratus* | 0.40 |
