# Supplementary material for: Community-led management maintains higher predator biomass supporting kelp forests persistence in Baja California
Source: Sci Rep. 2025 Jul 2;15:23253. doi: 10.1038/s41598-025-86140-6 (PMC12222674; doi:10.1038/s41598-025-86140-6)
Supplement: Supplementary file 2 — Supplementary Information 2. [file 41598_2025_86140_MOESM2_ESM.docx]

**Supplementary table 2.** Post-hoc comparison results of statistical analyzes (only significant differences shown) on the density (individuals per m^2^), size (cm), and biomass (kg) per transect of *S. pulcher*, *H. francisci*, and *P. interruptus*.

| Species | Measure | Comparison | p-value |
| --- | --- | --- | --- |
| *S. pulcher* | Density | North Inside - South Inside | 0.0644 |
|  |  | North Outside - South Inside | 0.0493 |
|  | Size | North Inside - South Inside | <0.0001 |
|  |  | North Outside - South Inside | 0.0004 |
|  |  | North Inside - South Outside | <0.0001 |
|  |  | North Outside - South Outside | <0.0001 |
|  | Biomass | North Inside - South Outside | 0.002 |
|  |  | North Outside - South Outside | 0.0014 |
| *H. francisci* | Density | North Inside - South Inside | 0.0177 |
|  |  | North Outside - South Inside | 0.0135 |
|  |  | North Inside - South Outside | 0.0328 |
|  |  | North Outside - South Outside | 0.0267 |
| *P. interruptus* | Density | North Inside - South Inside | <0.0001 |
|  |  | North Inside - North Outside | 0.0004 |
|  |  | North Inside - South Outside | <0.0001 |
|  |  | South Inside - North Outside | 0.0019 |
| *P. interruptus* | Biomass | North Inside - South Inside | <0.0001 |
|  |  | North Inside - North Outside | 0.0157 |
|  |  | North Inside - South Outside | 0.0001 |
|  |  | South Inside - North Outside | 0.0001 |
|  |  | South Inside - South outside | 0.0015 |
|  |  | North Outside - South Outside | 0.0561 |
